# Crystal structures of 1′-amino­cobaltocenium-1-carb­oxy­lic acid chloride monohydrate and of its azo dye 1′-[2-(1-amino-2,6-dimethylphenyl)diazen-1-yl]cobaltocenium-1-carb­oxy­lic acid hexa­fluorido­phosphate monohydrate

**DOI:** 10.1107/S2056989019000562

**Published:** 2019-01-15

**Authors:** Markus Jochriem, Klaus Wurst, Holger Kopacka, Benno Bildstein

**Affiliations:** a University of Innsbruck, Institute of General, Inorganic and Theoretical Chemistry, Center for Chemistry and Biomedicine, Innrain 80-82, 6020, Innsbruck, Austria

**Keywords:** crystal structure, cobaltocenium, azo dye, amino acid, metallocene, metallocenium, azo coupling

## Abstract

1′-Amino­cobaltocenium-1-carb­oxy­lic acid chloride, [Co(C_5_H_6_N)(C_6_H_5_O_2_)]Cl·H_2_O, (**3**), and its azo derivative 1′-[2-(1-amino-2,6-dimethylphenyl)diazen-1-yl]cobaltocenium-1-carb­oxy­lic acid hexa­fluorido­phosphate, [Co(C_13_H_14_N_3_)(C_6_H_5_O_2_)]PF_6_·H_2_O (**5**) were obtained from cobaltocenium-1,1′-di­carb­oxy­lic acid hexa­fluorido­phosphate by converting one carboxyl group to its chloro­carboxyl derivative followed by chloride/azide exchange, Curtius rearrangement, diazo­tiation and azo coupling with 2,6-di­methyl­aniline. Both title compounds crystallize as their monohydrates. In the crystal structure of **3**, both functional groups lie in the same direction, with the Cp rings being nearly eclipsed, and participate in an extended hydrogen-bonded supra­molecular network including the counter-ion and the water mol­ecule of crystallization. Although the functional groups in **5** are somewhat further apart, bearing a greater torsion angle with the Cp rings now staggered, a similar supra­molecular network is observed with not only the carb­oxy­lic acid and azo groups, but also with the more remote amino group participating in a hydrogen-bonded network, again including the counter-ion and the water mol­ecule. The hexa­fluorido­phosphate ion shows positional disorder. Compound **3** was refined as an inversion twin. In **5**, each of the six F atoms is disordered over two sets of sites in a 1:1 ratio.

## Abstract 2

Two asymmetrically substituted cobaltocenium carb­oxy­lic acid compounds were synthesized and their crystal structures determined. Both crystallize as hydrates and exhibit an extended hydrogen-bonding network.

## Chemical context   

**Figure 1:**
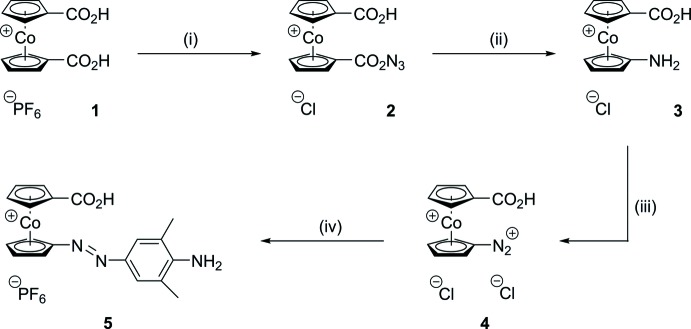
Synthetic scheme for obtaining the title compounds. (i) SOCl_2_/NaN_3_, (ii) H_2_SO_4_, (iii) HCl/NaNO_2_, (iv) 2,6-di­methyl­aniline.

One of the title compounds, 1′-amino­cobaltocenium-1-carb­oxy­lic acid chloride, **3**, is a new artificial organometallic amino acid. In comparison to its known iron analogue, 1′-amino­ferrocene-1-carb­oxy­lic acid (Butler & Quayle, 1998 ▸; Barišić *et al.*, 2002 ▸; Erb *et al.*, 2018 ▸) and its frequently studied bioorganometallic chemistry (Heinze & Schlenker, 2004 ▸; Heinze & Beckmann, 2005 ▸, 2007 ▸; Barišić *et al.* 2004 ▸, 2006**a* ▸,b* ▸, 2011 ▸, 2012 ▸; Mahmoud & Kraatz, 2007 ▸; Kovač *et al.*, 2009 ▸; Semenčić *et al.*, 2009 ▸; Semenčić *et al.*, 2010 ▸; Siebler *et al.*, 2010 ▸; Förster *et al.*, 2012 ▸; Kovačević *et al.*, 2014 ▸), 1′-amino­cobaltocenium-1-carb­oxy­lic acid chloride is an intrinsically cationic amino acid of similar potential in bioorganometallic peptide chemistry. Synthetically (Fig. 1 ▸), compound **3** was obtained from cobalto­cenium-1,1′-di­carb­oxy­lic acid hexa­fluorido­phosphate, **1** (Sheats & Rausch, 1970 ▸) in varying yields *via* Curtius rearrangement of its cobaltocenium-1′-carb­oxy­lic acid azide-1-carb­oxy­lic acid chloride, **2**, in analogy to our recent work on amino­cobaltocenium hexa­fluorido­phosphate (Vanicek *et al.*, 2016 ▸). The amino group of **3** was diazo­tized *in situ* with nitrous acid to yield 1′-diazo­nio-cobaltocenium-1-carb­oxy­lic acid dichloride, **4**, and reacted with 2,6-di­methyl­aniline to afford the new diazo dye 1′-[(diazene-1-yl)-2-(2,6-dimethyl-1-amino-phen-4-yl)]-cobaltocenium-1-carb­ox­y­lic acid hexa­fluorido­phosphate, **5**.
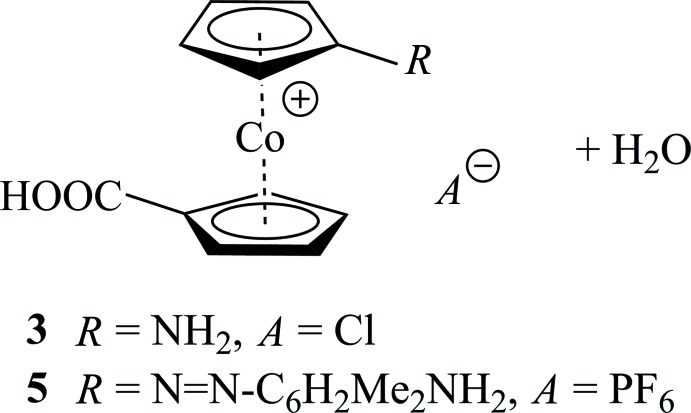


The mol­ecular and crystal structures of compounds **3** and **5** are reported in this communication.

## Structural commentary   

**Figure 2:**
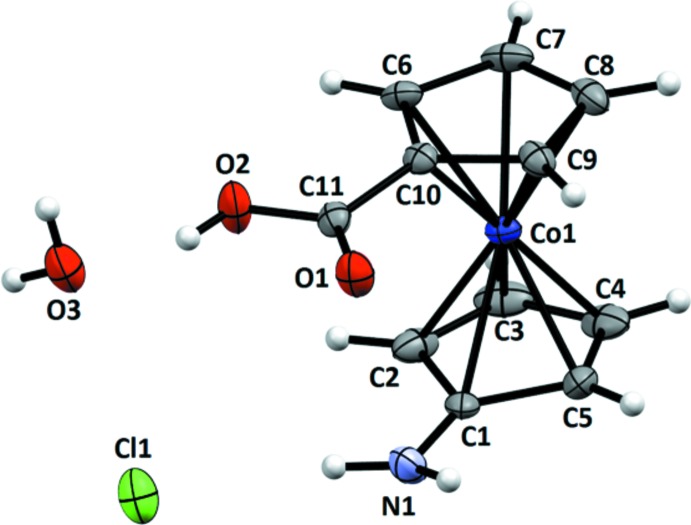
The mol­ecular entities in the structure of **3** with displacement ellipsoids for non-H atoms drawn at the 50% probability level.

Compounds **3** and **5** both crystallize as their monohydrates. Compound **3** forms crystals with one formula unit per asymmetric unit (Fig. 2 ▸). The cobalt atom is coordinated in a nearly eclipsed manner by the planar cyclo­penta­dienide rings with a torsion angle of 15° between the substituents, but the bond lengths between Co and C are not equal. In the carboxyl-substituted ring, the shortest distance [2.028 (3) Å] is found between Co1 and C10, the atom bearing the carboxyl group, as is to be expected from the electron-poorest carbon atom. Bond lengths involving the other four carbon atoms in this ring are considerably longer [Co—C_averaged_ = 2.052 Å]. On the other hand, in the amino-substituted ring, the N-bonded carbon atom C1 shows a significantly longer bond length [2.153 (3) Å] to Co1 than the other four carbon atoms in this ring [Co—C_averaged_ = 2.031]. In addition, the formal C—N single bond [C1—N1 = 1.343 (4) Å] of the amino substituent is considerably shortened, as has also been observed in amino­cobaltocenium tetra­phenyl­borate [C—N = 1.340 (3) Å; Vanicek *et al.*, 2016 ▸] and amino­penta­methyl­cobaltocenium hexa­fluorido­phosphate [C—N = 1.351 (5) Å; Wolter-Steingrube *et al.*, 2014 ▸]. This is caused by the contribution of a mesomeric structure featuring an η^4^-bound cyclo­penta­diene with an iminium group, a general effect observed in donor-substituted cobaltocenium salts (Sheats, 1979 ▸). The bond lengths and angles of the carboxyl substituent are unexceptional and in line with expectations.

**Figure 3:**
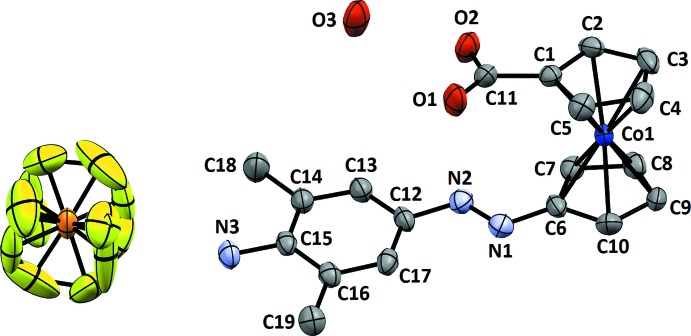
The mol­ecular entities in the structure of **5** with displacement ellipsoids drawn at the 50% probability level. Hydrogen atoms were omitted for clarity.

In the cobaltocenium cation of **5**, the cyclo­penta­dienide rings are almost staggered with the substituents oriented in roughly the same direction and a torsion angle of 29 (**s.u.?**)° (Fig. 3 ▸). The Co—C_ring_ distances show no great variation, with the exception being the bond to C6, *i.e.* the carbon atom connected to the azo group [2.064 (2) Å]. This bond is slightly elongated but not as much as the corresponding bond to the amino group in the structure of **3**. The azo group features a *trans*-configuration with distances typical for asymmetric azo compounds.

## Supra­molecular features   

**Figure 4:**
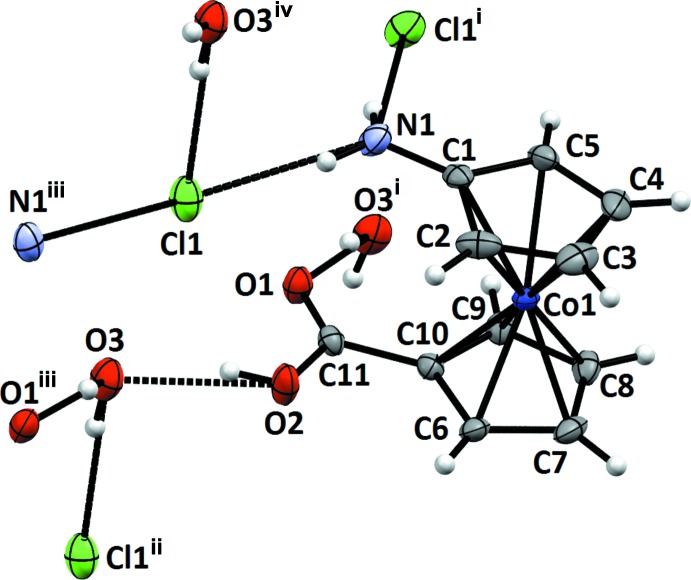
Hydrogen-bonding inter­actions between the amino group, the carboxyl group, the water mol­ecule of crystallization and the counter-anion in the crystal structure of **3**. Displacement ellipsoids as in Fig. 2 ▸. [Symmetry codes: (i) 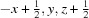; (ii) 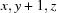; (iii) 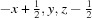.]

**Figure 5:**
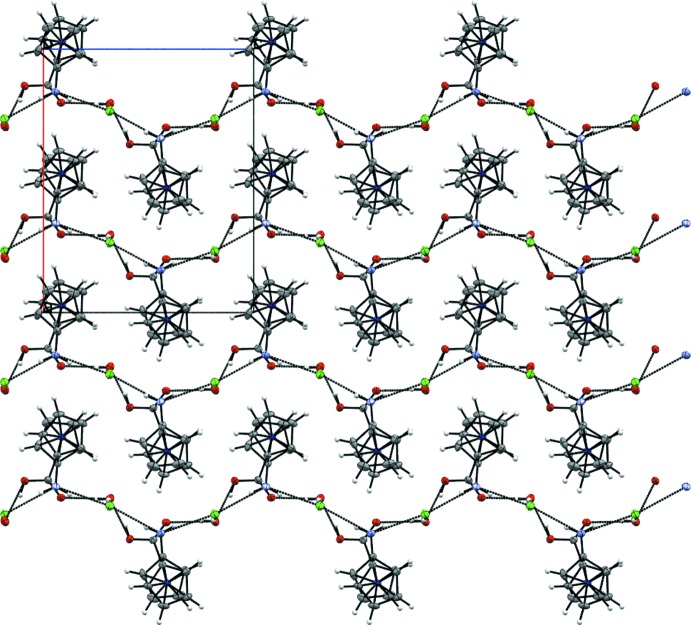
A view along the *b* axis of the crystal structure of **3** showing the formation of zigzag chains parallel to the *c* axis. Displacement ellipsoids as in Fig. 2 ▸.

**Figure 6:**
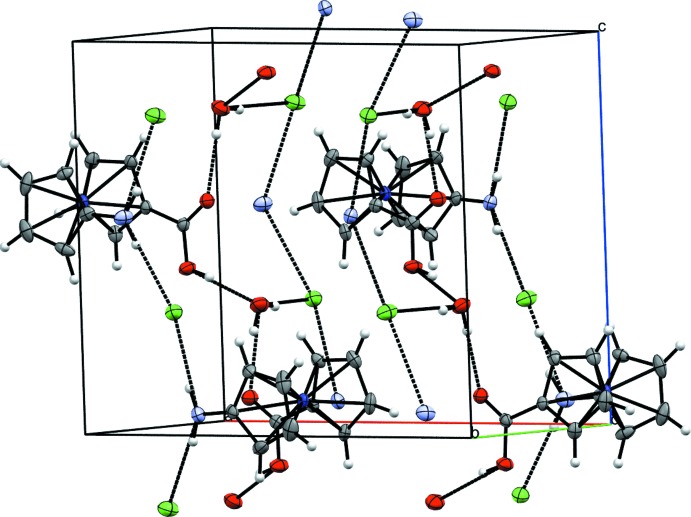
Ladder-type hydrogen-bonded network in the crystal structure of **3**. Displacement ellipsoids as in Fig. 2 ▸.

**Figure 7:**
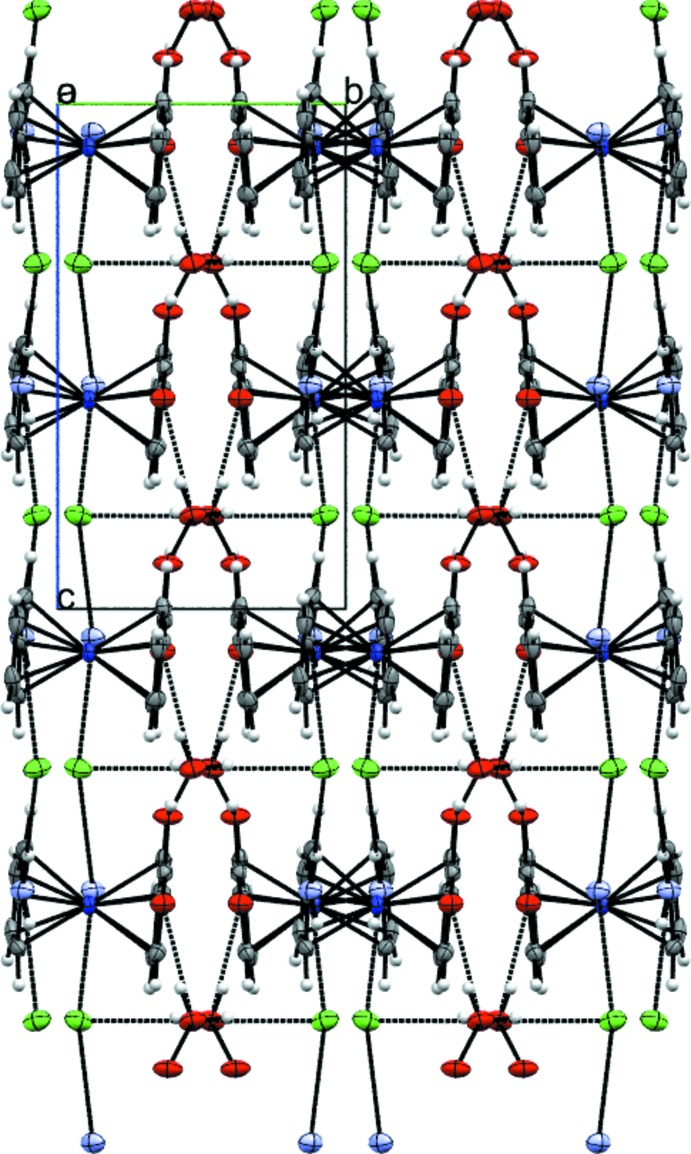
Formation of undulating layers parallel to (100) in the crystal structure of **3**. Displacement ellipsoids as in Fig. 2 ▸.

The water mol­ecule of crystallization, carboxyl group, amino group and chloride anion of **3** are part of an extended hydrogen-bonding network in the crystal (Fig. 4 ▸, Table 1 ▸). Zigzag chains are aligned parallel to the *c* axis (Fig. 5 ▸), in which every other mol­ecule shows the same orientation. These chains are formed by an infinite hydrogen-bonding network, comprised of water mol­ecules connecting the carboxyl groups of two neighboring cations and also forming a bond to the chloride anion. The chloride anions are also hydrogen-bonded to the NH_2_ groups of two more cations, therefore forming a ladder-type network in which the ladders are connected to each other by the cobaltocenium moieties (Fig. 6 ▸). Overall, this arrangement results in an undulating layer structure extending parallel to (100) (Fig. 7 ▸).

**Figure 8:**
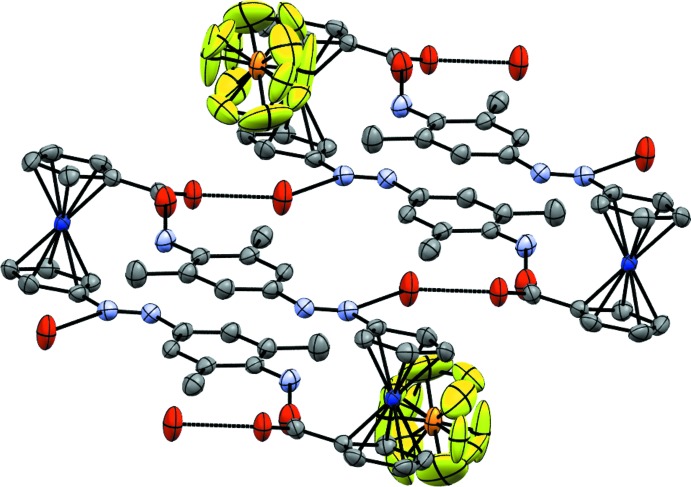
Formation of hydrogen-bonded dimers in the crystal structure of **5**. Displacement ellipsoids as in Fig. 3 ▸; hydrogen atoms were omitted for clarity.

**Figure 9:**
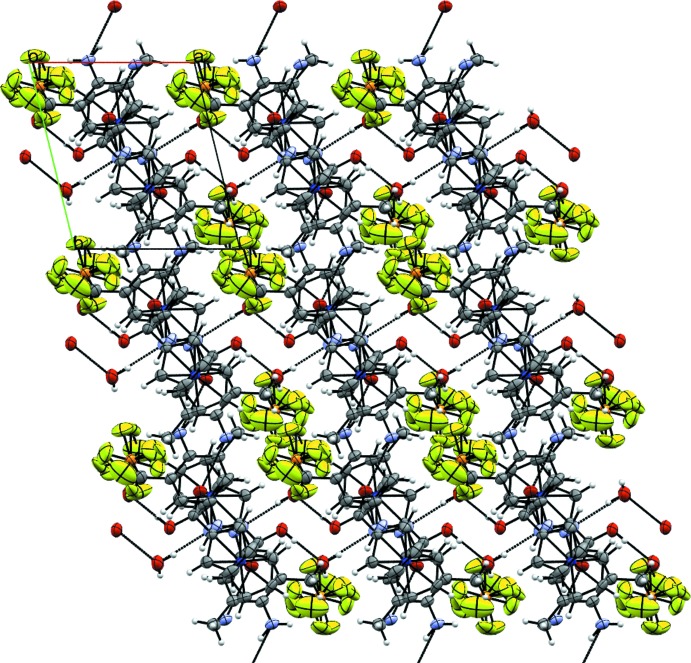
Mol­ecular packing of the crystal structure of **5** in a view along the *c* axis, showing the alternating anionic and cationic layers parallel to the *bc* plane. Displacement ellipsoids as in Fig. 3 ▸.

In the crystal structure of **5**, the azo, carboxyl, amino groups and the water mol­ecule of crystallization are part of a hydrogen-bonded network (Table 2 ▸). Dimers result from hydrogen bonds between the amino function (N3—H) of one mol­ecule and the carb­oxy­lic acid group (O1) of a neighbouring mol­ecule. Additionally, these dimers are connected to one another by water mol­ecules (O3), forming hydrogen bonds involving the carb­oxy­lic acid group (O1) and the azo group (N1). In addition, the disordered hexa­fluorido­phosphate ions inter­act with the otherwise unbound second hydrogen atom of the water mol­ecule and the second hydrogen atom of the amino functionality (Fig. 8 ▸), thereby forming layers parallel the *bc* plane that separate layers of cations (Fig. 9 ▸).

## Synthesis and crystallization   

Compound **3**: 1′-Amino­cobaltocenium-1-carb­oxy­lic acid chloride hydrate, **3**, was obtained in varying yields starting from cobaltocenium-1,1′-bis carb­oxy­lic acid hexa­fluorido­phosphate by converting it first to its mono carb­oxy­lic azide followed by Curtius rearrangement, in a variant analogous to monosubstituted cobaltocenium carb­oxy­lic acid hexa­fluorido­phosphate (Vanicek *et al.*, 2016 ▸). Column chroma­tography on alumina using methanol/water as eluent, separated it from 1,1′-di­amino­cobaltocenium, which was eluted before with aceto­nitrile. After addition of hydro­chloric acid to hydrolyze the meth­oxy­aluminum species, the volatiles were evaporated, the residue extracted with ethanol, filtered and dried first on a rotary evaporator and then *in vacuo*. Single crystals were obtained *via* slow concentration of a solution in methanol. ^1^H NMR (CD_3_OD), ppm: δ = 5.16 (pseudo-*t*, *J* = 2.1 Hz), 5.48 (pseudo-*t*, *J* = 2.1 Hz), 5.51 (pseudo-*t*, *J* = 2.1 Hz), 5.97 (pseudo-*t*, *J* = 2.1 Hz). ESI-MS showed a signal at 248.0139 *m*/*z* in accordance to the mol­ecular cation.

Compound **5**: 1′-Amino­cobaltocenium-1-carb­oxy­lic acid chloride hydrate (**3**) (100.9 mg, 0.3345 mmol, 1 equivalent) was dissolved in 5 ml of concentrated HCl and the mixture was cooled to 273 K. Then NaNO_2_ (26.6 mg, 0.3850 mmol, 1.15 equivalent) was added and the yellow solution was stirred for 15 min. After addition of 2,6-di­methyl­aniline (63.5 µl, 0.5134 mmol, 1.5 equivalents), the solution immediately turned red and was stirred for a further 30 min. When neutralized with saturated Na_2_CO_3_ solution, the reaction mixture again changed color to a darker red. The mixture was concentrated on a rotary evaporator and the salts were precipitated with ethanol. The solution was filtered, evaporated to dryness, the residue taken up in aceto­nitrile and after filtering and evaporating to dryness the product was dissolved in small amounts of water, and a few drops of aqueous HPF_6_ (60%) were added. The solution was extracted three times with di­chloro­methane, the combined dark-violet-colored organic phases were evaporated to dryness and the product (**5**) was dried *in vacuo*. Yield: 92.1 mg (52.2%) as a dark orange–red powder. Slow concentration of a solution in ethanol yielded single crystals suitable for X-ray analysis. ^1^H NMR (CD_3_OD), ppm: δ = 2.3 (2,6-Me, *t*, *J* = 0.6 Hz), 5.80 (pseudo-*t*, *J* = 2.1 Hz), 5.89 (pseudo-*t*, *J* = 2.1 Hz), 6.15 (pseudo-*t*, *J* = 2.1 Hz), 6.29 (pseudo-*t*, *J* = 2.1 Hz), 7.52 (3,5-CH, *t*, *J* = 0.6 Hz). ESI-MS showed a signal at 380.0836 *m*/*z* in accordance with the mol­ecular cation.

## Refinement   

Crystal data, data collection and structure refinement details are summarized in Table 3 ▸. In both compounds, C-bound H atoms were positioned geometrically (C—H = 0.95–0.98) and refined as riding with *U*_iso_(H) = 1.2*U*_eq_(C) or 1.5*U*_eq_(Cmeth­yl). For the refinement of **3**, H atoms bound to N1, O2 and O3 were found in difference-Fourier maps and were treated with restraints on bond lengths (*d* = 0.89 Å for N and *d* = 0.83 Å for O) and refined with isotropic displacement parameters. The crystal studied was refined as an inversion twin. For **5**, H atoms bound to N3 and O2 were treated in the same way as for **3** while the H atoms of the water mol­ecule (also found from a difference-Fourier map and treated with restraints on the bond length) were refined with *U*_iso_(H) = 1.2*U*_eq_(O3). The hexa­fluorido­phosphate ion shows positional disorder. Each of the six F atoms was refined with two sets of sites in a 1:1 ratio.

## Supplementary Material

Crystal structure: contains datablock(s) global, 3, 5. DOI: 10.1107/S2056989019000562/wm5478sup1.cif

Structure factors: contains datablock(s) 3. DOI: 10.1107/S2056989019000562/wm54783sup4.hkl

Structure factors: contains datablock(s) 5. DOI: 10.1107/S2056989019000562/wm54785sup5.hkl

CCDC references: 1884166, 1884167

Additional supporting information:  crystallographic information; 3D view; checkCIF report

## Figures and Tables

**Table 1:** Hydrogen-bond geometry (Å, °) for **3** [Chem scheme1]

*D*—H⋯*A*	*D*—H	H⋯*A*	*D*⋯*A*	*D*—H⋯*A*
O2—H2*O*⋯O3	0.82 (2)	1.78 (3)	2.577 (3)	163 (4)
N1—H1*N*⋯Cl1^i^	0.89 (2)	2.36 (3)	3.239 (3)	166 (4)
N1—H2*N*⋯Cl1	0.89 (2)	2.37 (3)	3.253 (3)	172 (3)
O3—H3*A*⋯Cl1^ii^	0.82 (2)	2.30 (2)	3.106 (3)	172 (4)
O3—H3*B*⋯O1^iii^	0.81 (2)	2.02 (3)	2.822 (4)	171 (3)

Symmetry codes: (i) 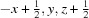; (ii) 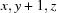; (iii) 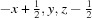.

**Table 2:** Hydrogen-bond geometry (Å, °) for **5** [Chem scheme1]

*D*—H⋯*A*	*D*—H	H⋯*A*	*D*⋯*A*	*D*—H⋯*A*
N3—H2*N*⋯O1^i^	0.88 (2)	2.18 (2)	3.015 (3)	159 (3)
N3—H1*N*⋯F5	0.88 (2)	2.29 (3)	2.994 (10)	137 (3)
N3—H1*N*⋯F5*A*	0.88 (2)	2.24 (3)	2.896 (8)	131 (3)
O2—H2*O*⋯O3	0.84 (2)	1.80 (2)	2.625 (3)	170 (4)
O3—H3*A*⋯N1^ii^	0.86 (2)	2.06 (2)	2.907 (3)	171 (4)
O3—H3*B*⋯F5^iii^	0.84 (2)	2.22 (3)	2.988 (8)	153 (4)
O3—H3*B*⋯F2*A* ^iii^	0.84 (2)	2.34 (3)	3.112 (8)	154 (4)

Symmetry codes: (i) 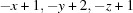; (ii) 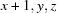; (iii) 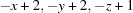.

**Table 3:** Experimental details

	**3**	**5**
Crystal data		
Chemical formula	[Co(C_6_H_5_N)(C_6_H_5_O_2_)]Cl·H_2_O	[Co(C_6_H_5_O_2_)]PF_6_·H_2_O
*M*_r_	301.60	543.29
Crystal system, space group	Orthorhombic, *P* *c* *a*2_1_	Triclinic, *P* 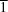
Temperature (K)	193	191
*a*, *b*, *c* (Å)	14.7269 (5), 6.7024 (3), 11.7607 (4)	7.9891 (4), 9.4310 (5), 15.5425 (8)
α, β, γ (°)	90, 90, 90	74.415 (3), 78.183 (2), 73.798 (2)
*V* (Å^3^)	1160.85 (8)	1072.48 (10)
*Z*	4	2
Radiation type	Mo *K*α	Mo *K*α
μ (mm^−1^)	1.70	0.95
Crystal size (mm)	0.13 × 0.11 × 0.03	0.16 × 0.16 × 0.03

Data collection		
Diffractometer	Bruker D8 QUEST PHOTON 100	Bruker D8 QUEST PHOTON 100
Absorption correction	Multi-scan (*SADABS*; Krause *et al.*, 2015 ▸)	Multi-scan (*SADABS*; Krause *et al.*, 2015 ▸)
*T*_min_, *T*_max_	0.858, 0.942	0.826, 0.901
No. of measured, independent and observed [*I* > 2σ(*I*)] reflections	14289, 2163, 2099	20686, 3945, 3290
*R*_int_	0.031	0.043

Refinement		
*R*[*F* ^2^ > 2σ(*F* ^2^)], *wR*(*F* ^2^), *S*	0.020, 0.046, 1.08	0.035, 0.086, 1.04
No. of reflections	2163	3945
No. of parameters	175	372
No. of restraints	6	5
H-atom treatment	H atoms treated by a mixture of independent and constrained refinement	H atoms treated by a mixture of independent and constrained refinement
Δρ_max_, Δρ_min_ (e Å^−3^)	0.50, −0.31	0.54, −0.31
Absolute structure	Refined as an inversion twin	–
Absolute structure parameter	0.067 (17)	–

Computer programs: *APEX3* and *SAINT* (Bruker, 2013 ▸), *SHELXT* (Sheldrick, 2015*a* ▸), *SHELXL* (Sheldrick, 2015*b* ▸), *CHEMDRAW* (Cambridge Soft, 2001 ▸), *ORTEP-3 for Windows* (Farrugia, 2012 ▸) and *publCIF* (Westrip, 2010 ▸).

## supplementary crystallographic information

### 1'-Aminocobaltocenium-1-carboxylic acid chloride monohydrate (3) Crystal data

**Table:**

[Co(C_5_H_6_N)(C_6_H_5_O_2_)]Cl·H_2_O	*D*_x_ = 1.726 Mg m^−^^3^
*M**_r_* = 301.60	Mo *K*α radiation, λ = 0.71073 Å
Orthorhombic, *P**c**a*2_1_	Cell parameters from 9295 reflections
*a* = 14.7269 (5) Å	θ = 2.7–50.7°
*b* = 6.7024 (3) Å	µ = 1.70 mm^−^^1^
*c* = 11.7607 (4) Å	*T* = 193 K
*V* = 1160.85 (8) Å^3^	Plate, orange
*Z* = 4	0.13 × 0.11 × 0.03 mm
*F*(000) = 616	

### 1'-Aminocobaltocenium-1-carboxylic acid chloride monohydrate (3) Data collection

**Table:**

Bruker D8 QUEST PHOTON 100 diffractometer	2163 independent reflections
Radiation source: Incoatec Microfocus	2099 reflections with *I* > 2σ(*I*)
Multi layered optics monochromator	*R*_int_ = 0.031
Detector resolution: 10.4 pixels mm^-1^	θ_max_ = 25.7°, θ_min_ = 2.8°
φ and ω scans	*h* = −17→17
Absorption correction: multi-scan (*SADABS*; Krause *et al.*, 2015)	*k* = −8→8
*T*_min_ = 0.858, *T*_max_ = 0.942	*l* = −14→13
14289 measured reflections	

### 1'-Aminocobaltocenium-1-carboxylic acid chloride monohydrate (3) Refinement

**Table:**

Refinement on *F*^2^	Hydrogen site location: mixed
Least-squares matrix: full	H atoms treated by a mixture of independent and constrained refinement
*R*[*F*^2^ > 2σ(*F*^2^)] = 0.020	*w* = 1/[σ^2^(*F*_o_^2^) + (0.0262*P*)^2^ + 0.115*P*] where *P* = (*F*_o_^2^ + 2*F*_c_^2^)/3
*wR*(*F*^2^) = 0.046	(Δ/σ)_max_ = 0.001
*S* = 1.08	Δρ_max_ = 0.50 e Å^−^^3^
2163 reflections	Δρ_min_ = −0.31 e Å^−^^3^
175 parameters	Absolute structure: Refined as an inversion twin
6 restraints	Absolute structure parameter: 0.067 (17)

### 1'-Aminocobaltocenium-1-carboxylic acid chloride monohydrate (3) Special details

**Table:**

Geometry. All esds (except the esd in the dihedral angle between two l.s. planes) are estimated using the full covariance matrix. The cell esds are taken into account individually in the estimation of esds in distances, angles and torsion angles; correlations between esds in cell parameters are only used when they are defined by crystal symmetry. An approximate (isotropic) treatment of cell esds is used for estimating esds involving l.s. planes.
Refinement. Refined as a two-component inversion twin. Hydrogens at N1, O2 and O3 were found and refined isotropically with bond restraints (d = 89 pm for N and d = 83 pm for O).

### 1'-Aminocobaltocenium-1-carboxylic acid chloride monohydrate (3) Fractional atomic coordinates and isotropic or equivalent isotropic displacement parameters (Å^2^)

**Table:**

	*x*	*y*	*z*	*U*_iso_*/*U*_eq_	
Co1	0.52686 (2)	0.10826 (4)	0.58678 (3)	0.01564 (11)	
Cl1	0.23429 (5)	−0.07147 (13)	0.31541 (7)	0.0317 (2)	
O1	0.29710 (11)	0.3651 (3)	0.5834 (2)	0.0246 (4)	
O2	0.36058 (15)	0.3830 (4)	0.41056 (19)	0.0286 (5)	
H2O	0.3077 (19)	0.393 (6)	0.389 (4)	0.037 (12)*	
N1	0.33888 (18)	−0.1222 (4)	0.5573 (2)	0.0256 (6)	
H1N	0.310 (3)	−0.099 (5)	0.623 (2)	0.041 (12)*	
H2N	0.315 (2)	−0.102 (5)	0.489 (2)	0.035 (10)*	
C1	0.42978 (19)	−0.1291 (4)	0.5660 (2)	0.0180 (6)	
C2	0.4952 (3)	−0.1153 (5)	0.4754 (3)	0.0262 (8)	
H2	0.4817	−0.0925	0.3975	0.031*	
C3	0.5837 (3)	−0.1416 (6)	0.5224 (4)	0.0307 (9)	
H3	0.6390	−0.1480	0.4810	0.037*	
C4	0.5743 (2)	−0.1564 (5)	0.6425 (3)	0.0301 (8)	
H4	0.6225	−0.1746	0.6953	0.036*	
C5	0.4807 (2)	−0.1393 (5)	0.6701 (3)	0.0212 (8)	
H5	0.4560	−0.1352	0.7447	0.025*	
C6	0.5386 (2)	0.3681 (4)	0.4969 (3)	0.0191 (6)	
H6	0.5413	0.3791	0.4164	0.023*	
C7	0.61365 (18)	0.3494 (4)	0.5717 (3)	0.0249 (7)	
H7	0.6757	0.3472	0.5499	0.030*	
C8	0.5806 (2)	0.3345 (5)	0.6843 (3)	0.0228 (6)	
H8	0.6166	0.3198	0.7509	0.027*	
C9	0.4845 (2)	0.3453 (5)	0.6807 (3)	0.0194 (6)	
H9	0.4449	0.3391	0.7443	0.023*	
C10	0.45771 (18)	0.3671 (4)	0.5654 (2)	0.0161 (7)	
C11	0.3632 (2)	0.3719 (4)	0.5221 (2)	0.0176 (6)	
O3	0.20765 (15)	0.4689 (4)	0.3159 (2)	0.0312 (5)	
H3A	0.212 (3)	0.590 (4)	0.309 (4)	0.036 (12)*	
H3B	0.210 (2)	0.428 (5)	0.251 (2)	0.024 (10)*	

### 1'-Aminocobaltocenium-1-carboxylic acid chloride monohydrate (3) Atomic displacement parameters (Å^2^)

**Table:**

	*U*^11^	*U*^22^	*U*^33^	*U*^12^	*U*^13^	*U*^23^
Co1	0.01400 (17)	0.01243 (17)	0.02049 (19)	−0.00030 (13)	0.00119 (18)	0.0009 (2)
Cl1	0.0290 (5)	0.0424 (4)	0.0238 (4)	0.0066 (3)	−0.0033 (3)	−0.0045 (3)
O1	0.0177 (8)	0.0342 (10)	0.0219 (9)	0.0028 (8)	0.0008 (12)	−0.0004 (11)
O2	0.0193 (11)	0.0487 (15)	0.0177 (11)	0.0029 (10)	−0.0022 (9)	0.0023 (9)
N1	0.0211 (13)	0.0325 (15)	0.0231 (16)	−0.0081 (11)	−0.0023 (10)	−0.0003 (10)
C1	0.0234 (14)	0.0120 (12)	0.0187 (18)	−0.0042 (10)	−0.0029 (12)	0.0002 (11)
C2	0.0400 (18)	0.0180 (17)	0.0205 (17)	−0.0060 (14)	0.0091 (16)	−0.0037 (13)
C3	0.0271 (19)	0.0152 (18)	0.050 (2)	0.0015 (14)	0.0104 (16)	−0.0045 (15)
C4	0.0236 (18)	0.0145 (17)	0.052 (2)	0.0019 (15)	−0.0080 (16)	0.0072 (16)
C5	0.0260 (18)	0.0177 (17)	0.0197 (18)	−0.0040 (12)	−0.0021 (13)	0.0044 (12)
C6	0.0158 (15)	0.0155 (16)	0.0259 (16)	−0.0028 (12)	0.0032 (12)	0.0020 (12)
C7	0.0152 (12)	0.0158 (12)	0.044 (2)	−0.0029 (10)	0.0041 (15)	0.0010 (15)
C8	0.0207 (15)	0.0180 (14)	0.0296 (16)	0.0001 (13)	−0.0077 (12)	−0.0042 (12)
C9	0.0195 (16)	0.0169 (14)	0.0218 (16)	0.0020 (12)	−0.0032 (12)	−0.0038 (12)
C10	0.0168 (12)	0.0128 (12)	0.019 (2)	0.0016 (10)	0.0002 (12)	−0.0009 (11)
C11	0.0195 (14)	0.0149 (14)	0.0186 (15)	0.0029 (11)	−0.0021 (11)	0.0001 (10)
O3	0.0283 (12)	0.0415 (16)	0.0237 (12)	−0.0009 (11)	−0.0054 (11)	−0.0047 (12)

### 1'-Aminocobaltocenium-1-carboxylic acid chloride monohydrate (3) Geometric parameters (Å, º)

**Table:**

Co1—C4	2.016 (4)	C2—H2	0.9500
Co1—C3	2.019 (4)	C3—C4	1.423 (5)
Co1—C10	2.028 (3)	C3—H3	0.9500
Co1—C9	2.033 (3)	C4—C5	1.421 (5)
Co1—C5	2.043 (3)	C4—H4	0.9500
Co1—C2	2.044 (4)	C5—H5	0.9500
Co1—C6	2.044 (3)	C6—C7	1.418 (4)
Co1—C8	2.060 (3)	C6—C10	1.439 (4)
Co1—C7	2.068 (3)	C6—H6	0.9500
Co1—C1	2.153 (3)	C7—C8	1.414 (5)
O1—C11	1.213 (4)	C7—H7	0.9500
O2—C11	1.314 (4)	C8—C9	1.419 (4)
O2—H2O	0.82 (2)	C8—H8	0.9500
N1—C1	1.343 (4)	C9—C10	1.419 (4)
N1—H1N	0.89 (2)	C9—H9	0.9500
N1—H2N	0.89 (2)	C10—C11	1.482 (4)
C1—C5	1.437 (4)	O3—H3A	0.82 (2)
C1—C2	1.439 (5)	O3—H3B	0.81 (2)
C2—C3	1.427 (6)		
			
C4—Co1—C3	41.29 (13)	C1—C2—Co1	74.07 (18)
C4—Co1—C10	165.32 (14)	C3—C2—H2	125.8
C3—Co1—C10	150.63 (14)	C1—C2—H2	125.8
C4—Co1—C9	128.13 (14)	Co1—C2—H2	123.2
C3—Co1—C9	168.22 (15)	C4—C3—C2	107.7 (4)
C10—Co1—C9	40.91 (12)	C4—C3—Co1	69.2 (2)
C4—Co1—C5	40.98 (16)	C2—C3—Co1	70.4 (2)
C3—Co1—C5	69.17 (14)	C4—C3—H3	126.1
C10—Co1—C5	125.96 (13)	C2—C3—H3	126.1
C9—Co1—C5	105.78 (15)	Co1—C3—H3	125.8
C4—Co1—C2	69.06 (16)	C5—C4—C3	108.4 (4)
C3—Co1—C2	41.12 (17)	C5—C4—Co1	70.53 (19)
C10—Co1—C2	115.67 (14)	C3—C4—Co1	69.5 (2)
C9—Co1—C2	148.29 (15)	C5—C4—H4	125.8
C5—Co1—C2	68.66 (15)	C3—C4—H4	125.8
C4—Co1—C6	152.63 (14)	Co1—C4—H4	125.8
C3—Co1—C6	118.50 (14)	C4—C5—C1	108.4 (3)
C10—Co1—C6	41.38 (11)	C4—C5—Co1	68.49 (18)
C9—Co1—C6	68.98 (12)	C1—C5—Co1	74.15 (18)
C5—Co1—C6	165.42 (13)	C4—C5—H5	125.8
C2—Co1—C6	108.20 (14)	C1—C5—H5	125.8
C4—Co1—C8	109.46 (15)	Co1—C5—H5	123.2
C3—Co1—C8	131.28 (15)	C7—C6—C10	107.3 (3)
C10—Co1—C8	68.42 (12)	C7—C6—Co1	70.72 (17)
C9—Co1—C8	40.56 (12)	C10—C6—Co1	68.68 (16)
C5—Co1—C8	117.29 (15)	C7—C6—H6	126.4
C2—Co1—C8	169.83 (14)	C10—C6—H6	126.4
C6—Co1—C8	68.19 (12)	Co1—C6—H6	125.8
C4—Co1—C7	120.07 (14)	C8—C7—C6	108.7 (3)
C3—Co1—C7	111.08 (14)	C8—C7—Co1	69.65 (17)
C10—Co1—C7	68.35 (11)	C6—C7—Co1	68.94 (16)
C9—Co1—C7	68.01 (13)	C8—C7—H7	125.7
C5—Co1—C7	151.79 (13)	C6—C7—H7	125.7
C2—Co1—C7	131.23 (15)	Co1—C7—H7	127.3
C6—Co1—C7	40.34 (13)	C7—C8—C9	108.1 (3)
C8—Co1—C7	40.07 (14)	C7—C8—Co1	70.27 (17)
C4—Co1—C1	67.48 (13)	C9—C8—Co1	68.71 (17)
C3—Co1—C1	67.67 (13)	C7—C8—H8	125.9
C10—Co1—C1	106.55 (11)	C9—C8—H8	125.9
C9—Co1—C1	115.79 (11)	Co1—C8—H8	126.6
C5—Co1—C1	39.94 (12)	C8—C9—C10	108.1 (3)
C2—Co1—C1	40.01 (13)	C8—C9—Co1	70.73 (17)
C6—Co1—C1	128.84 (12)	C10—C9—Co1	69.34 (17)
C8—Co1—C1	149.60 (12)	C8—C9—H9	125.9
C7—Co1—C1	168.03 (13)	C10—C9—H9	125.9
C11—O2—H2O	110 (3)	Co1—C9—H9	125.6
C1—N1—H1N	114 (3)	C9—C10—C6	107.8 (2)
C1—N1—H2N	118 (2)	C9—C10—C11	126.3 (3)
H1N—N1—H2N	125 (4)	C6—C10—C11	125.8 (3)
N1—C1—C5	125.9 (3)	C9—C10—Co1	69.76 (16)
N1—C1—C2	127.4 (3)	C6—C10—Co1	69.94 (16)
C5—C1—C2	106.5 (3)	C11—C10—Co1	122.18 (18)
N1—C1—Co1	130.2 (2)	O1—C11—O2	124.9 (3)
C5—C1—Co1	65.91 (16)	O1—C11—C10	123.3 (3)
C2—C1—Co1	65.92 (18)	O2—C11—C10	111.8 (3)
C3—C2—C1	108.5 (3)	H3A—O3—H3B	104 (4)
C3—C2—Co1	68.5 (2)		
			
N1—C1—C2—C3	−176.6 (3)	C6—C7—C8—C9	−0.4 (3)
C5—C1—C2—C3	7.0 (3)	Co1—C7—C8—C9	−58.5 (2)
Co1—C1—C2—C3	60.2 (2)	C6—C7—C8—Co1	58.0 (2)
N1—C1—C2—Co1	123.2 (3)	C7—C8—C9—C10	0.0 (3)
C5—C1—C2—Co1	−53.2 (2)	Co1—C8—C9—C10	−59.5 (2)
C1—C2—C3—C4	−4.4 (4)	C7—C8—C9—Co1	59.4 (2)
Co1—C2—C3—C4	59.4 (3)	C8—C9—C10—C6	0.5 (3)
C1—C2—C3—Co1	−63.7 (2)	Co1—C9—C10—C6	−59.84 (19)
C2—C3—C4—C5	−0.1 (5)	C8—C9—C10—C11	176.0 (2)
Co1—C3—C4—C5	60.0 (3)	Co1—C9—C10—C11	115.7 (3)
C2—C3—C4—Co1	−60.1 (3)	C8—C9—C10—Co1	60.3 (2)
C3—C4—C5—C1	4.5 (4)	C7—C6—C10—C9	−0.8 (3)
Co1—C4—C5—C1	63.9 (2)	Co1—C6—C10—C9	59.7 (2)
C3—C4—C5—Co1	−59.4 (3)	C7—C6—C10—C11	−176.3 (2)
N1—C1—C5—C4	176.5 (3)	Co1—C6—C10—C11	−115.8 (3)
C2—C1—C5—C4	−7.0 (3)	C7—C6—C10—Co1	−60.49 (19)
Co1—C1—C5—C4	−60.2 (2)	C9—C10—C11—O1	3.0 (4)
N1—C1—C5—Co1	−123.3 (3)	C6—C10—C11—O1	177.8 (3)
C2—C1—C5—Co1	53.22 (19)	Co1—C10—C11—O1	90.4 (3)
C10—C6—C7—C8	0.7 (3)	C9—C10—C11—O2	−176.6 (3)
Co1—C6—C7—C8	−58.5 (2)	C6—C10—C11—O2	−1.8 (4)
C10—C6—C7—Co1	59.19 (19)	Co1—C10—C11—O2	−89.2 (3)

### 1'-Aminocobaltocenium-1-carboxylic acid chloride monohydrate (3) Hydrogen-bond geometry (Å, º)

**Table:**

*D*—H···*A*	*D*—H	H···*A*	*D*···*A*	*D*—H···*A*
O2—H2*O*···O3	0.82 (2)	1.78 (3)	2.577 (3)	163 (4)
N1—H1*N*···Cl1^i^	0.89 (2)	2.36 (3)	3.239 (3)	166 (4)
N1—H2*N*···Cl1	0.89 (2)	2.37 (3)	3.253 (3)	172 (3)
O3—H3*A*···Cl1^ii^	0.82 (2)	2.30 (2)	3.106 (3)	172 (4)
O3—H3*B*···O1^iii^	0.81 (2)	2.02 (3)	2.822 (4)	171 (3)

Symmetry codes: (i) −*x*+1/2, *y*, *z*+1/2; (ii) *x*, *y*+1, *z*; (iii) −*x*+1/2, *y*, *z*−1/2.

### 1'-[2-(1-Amino-2,6-dimethylphenyl)diazen-1-yl]cobaltocenium-1-carboxylic acid hexafluoridophosphate monohydrate (5) Crystal data

**Table:**

[Co(C_13_H_14_N_3_)(C_6_H_5_O_2_)]PF_6_·H_2_O	*Z* = 2
*M**_r_* = 543.29	*F*(000) = 552
Triclinic, *P*1	*D*_x_ = 1.682 Mg m^−^^3^
*a* = 7.9891 (4) Å	Mo *K*α radiation, λ = 0.71073 Å
*b* = 9.4310 (5) Å	Cell parameters from 8266 reflections
*c* = 15.5425 (8) Å	θ = 2.3–25.3°
α = 74.415 (3)°	µ = 0.95 mm^−^^1^
β = 78.183 (2)°	*T* = 191 K
γ = 73.798 (2)°	Plate, brown
*V* = 1072.48 (10) Å^3^	0.16 × 0.16 × 0.03 mm

### 1'-[2-(1-Amino-2,6-dimethylphenyl)diazen-1-yl]cobaltocenium-1-carboxylic acid hexafluoridophosphate monohydrate (5) Data collection

**Table:**

Bruker D8 QUEST PHOTON 100 diffractometer	3945 independent reflections
Radiation source: Incoatec Microfocus	3290 reflections with *I* > 2σ(*I*)
Multi layered optics monochromator	*R*_int_ = 0.043
Detector resolution: 10.4 pixels mm^-1^	θ_max_ = 25.4°, θ_min_ = 2.3°
φ and ω scans	*h* = −9→9
Absorption correction: multi-scan (*SADABS*; Krause *et al.*, 2015)	*k* = −11→11
*T*_min_ = 0.826, *T*_max_ = 0.901	*l* = −18→18
20686 measured reflections	

### 1'-[2-(1-Amino-2,6-dimethylphenyl)diazen-1-yl]cobaltocenium-1-carboxylic acid hexafluoridophosphate monohydrate (5) Refinement

**Table:**

Refinement on *F*^2^	5 restraints
Least-squares matrix: full	Hydrogen site location: mixed
*R*[*F*^2^ > 2σ(*F*^2^)] = 0.035	H atoms treated by a mixture of independent and constrained refinement
*wR*(*F*^2^) = 0.086	*w* = 1/[σ^2^(*F*_o_^2^) + (0.0353*P*)^2^ + 0.9559*P*] where *P* = (*F*_o_^2^ + 2*F*_c_^2^)/3
*S* = 1.04	(Δ/σ)_max_ = 0.001
3945 reflections	Δρ_max_ = 0.54 e Å^−^^3^
372 parameters	Δρ_min_ = −0.31 e Å^−^^3^

### 1'-[2-(1-Amino-2,6-dimethylphenyl)diazen-1-yl]cobaltocenium-1-carboxylic acid hexafluoridophosphate monohydrate (5) Special details

**Table:**

Geometry. All esds (except the esd in the dihedral angle between two l.s. planes) are estimated using the full covariance matrix. The cell esds are taken into account individually in the estimation of esds in distances, angles and torsion angles; correlations between esds in cell parameters are only used when they are defined by crystal symmetry. An approximate (isotropic) treatment of cell esds is used for estimating esds involving l.s. planes.
Refinement. Hydrogen atoms at N3 and O2 were found and refined isotropically with bond restraints (d=89pm for N and d=83pm for). Also the hydrogens at water molecule were found, refined with bond restraints but with isotropic displacement parameter of 1.2 higher than U(iso) of O3. The flourine of the anion PF6- show a nearly 1:1 positional disorder F1-F1: F1A-F6A.

### 1'-[2-(1-Amino-2,6-dimethylphenyl)diazen-1-yl]cobaltocenium-1-carboxylic acid hexafluoridophosphate monohydrate (5) Fractional atomic coordinates and isotropic or equivalent isotropic displacement parameters (Å^2^)

**Table:**

	*x*	*y*	*z*	*U*_iso_*/*U*_eq_	Occ. (<1)
Co1	0.45960 (4)	0.32404 (4)	0.84877 (2)	0.02519 (11)	
O1	0.6004 (3)	0.7001 (2)	0.75554 (15)	0.0513 (6)	
O2	0.8374 (3)	0.5069 (2)	0.75963 (15)	0.0469 (5)	
H2O	0.895 (5)	0.564 (4)	0.722 (2)	0.091 (14)*	
N1	0.3994 (3)	0.5104 (3)	0.65242 (15)	0.0361 (5)	
N2	0.5502 (3)	0.5139 (3)	0.60815 (14)	0.0352 (5)	
N3	0.6392 (4)	1.0108 (3)	0.33140 (16)	0.0381 (5)	
H1N	0.746 (3)	1.013 (4)	0.303 (2)	0.057 (10)*	
H2N	0.549 (3)	1.087 (3)	0.317 (2)	0.057 (10)*	
C1	0.5893 (3)	0.4716 (3)	0.86280 (16)	0.0280 (5)	
C2	0.6649 (4)	0.3213 (3)	0.90866 (18)	0.0350 (6)	
H2	0.7831	0.2661	0.8968	0.042*	
C3	0.5322 (4)	0.2695 (3)	0.97471 (18)	0.0449 (7)	
H3	0.5459	0.1732	1.0155	0.054*	
C4	0.3756 (4)	0.3851 (4)	0.96977 (18)	0.0462 (8)	
H4	0.2657	0.3796	1.0064	0.055*	
C5	0.4106 (4)	0.5106 (3)	0.90090 (18)	0.0366 (6)	
H5	0.3287	0.6042	0.8834	0.044*	
C6	0.4099 (4)	0.3705 (3)	0.71763 (16)	0.0327 (6)	
C7	0.5527 (4)	0.2416 (3)	0.73550 (17)	0.0339 (6)	
H7	0.6701	0.2312	0.7053	0.041*	
C8	0.4880 (4)	0.1317 (3)	0.80667 (18)	0.0379 (6)	
H8	0.5542	0.0336	0.8314	0.046*	
C9	0.3095 (4)	0.1925 (3)	0.83431 (18)	0.0403 (7)	
H9	0.2345	0.1427	0.8811	0.048*	
C10	0.2603 (4)	0.3404 (3)	0.78064 (18)	0.0390 (6)	
H10	0.1473	0.4078	0.7857	0.047*	
C11	0.6753 (3)	0.5731 (3)	0.78691 (18)	0.0330 (6)	
C12	0.5566 (4)	0.6439 (3)	0.53849 (16)	0.0326 (6)	
C13	0.7250 (4)	0.6491 (3)	0.49348 (17)	0.0351 (6)	
H13	0.8212	0.5671	0.5109	0.042*	
C14	0.7569 (4)	0.7695 (3)	0.42443 (17)	0.0350 (6)	
C15	0.6122 (3)	0.8902 (3)	0.39860 (16)	0.0310 (6)	
C16	0.4380 (3)	0.8863 (3)	0.44206 (16)	0.0316 (6)	
C17	0.4144 (4)	0.7632 (3)	0.51119 (16)	0.0345 (6)	
H17	0.2988	0.7593	0.5410	0.041*	
C18	0.9404 (4)	0.7752 (4)	0.3787 (2)	0.0503 (8)	
H18A	1.0240	0.6850	0.4065	0.076*	
H18B	0.9683	0.8662	0.3854	0.076*	
H18C	0.9486	0.7782	0.3145	0.076*	
C19	0.2862 (4)	1.0137 (3)	0.41259 (19)	0.0406 (7)	
H19A	0.1771	0.9950	0.4507	0.061*	
H19B	0.2775	1.0203	0.3496	0.061*	
H19C	0.3046	1.1091	0.4182	0.061*	
P1	1.02857 (10)	1.13338 (9)	0.12566 (5)	0.0440 (2)	
F1	0.8815 (9)	1.0964 (8)	0.0890 (6)	0.108 (2)	0.5
F2	1.1759 (6)	1.1734 (6)	0.1606 (5)	0.0803 (14)	0.5
F3	0.9908 (6)	1.2937 (6)	0.0619 (6)	0.090 (2)	0.5
F4	1.0704 (9)	0.9680 (7)	0.1867 (4)	0.088 (3)	0.5
F5	0.8818 (13)	1.1883 (11)	0.2032 (6)	0.083 (3)	0.5
F6	1.1786 (15)	1.0780 (13)	0.0534 (6)	0.105 (4)	0.5
F1A	0.9618 (12)	1.1992 (12)	0.0352 (5)	0.142 (3)	0.5
F2A	1.0947 (12)	1.0805 (10)	0.2208 (4)	0.141 (3)	0.5
F3A	1.0275 (11)	1.3037 (7)	0.1226 (5)	0.113 (3)	0.5
F4A	1.0215 (11)	0.9684 (8)	0.1367 (7)	0.118 (3)	0.5
F5A	0.8398 (14)	1.1641 (10)	0.1748 (9)	0.179 (7)	0.5
F6A	1.2124 (13)	1.0946 (12)	0.0709 (8)	0.138 (6)	0.5
O3	1.0363 (3)	0.6853 (3)	0.65768 (18)	0.0613 (7)	
H3A	1.145 (3)	0.639 (4)	0.650 (2)	0.074*	
H3B	1.037 (5)	0.747 (4)	0.687 (2)	0.074*	

### 1'-[2-(1-Amino-2,6-dimethylphenyl)diazen-1-yl]cobaltocenium-1-carboxylic acid hexafluoridophosphate monohydrate (5) Atomic displacement parameters (Å^2^)

**Table:**

	*U*^11^	*U*^22^	*U*^33^	*U*^12^	*U*^13^	*U*^23^
Co1	0.02688 (19)	0.02523 (18)	0.02200 (18)	−0.00943 (13)	−0.00294 (12)	0.00010 (13)
O1	0.0437 (12)	0.0342 (12)	0.0620 (14)	−0.0097 (9)	−0.0085 (10)	0.0136 (10)
O2	0.0301 (11)	0.0478 (12)	0.0540 (13)	−0.0124 (9)	0.0035 (9)	−0.0004 (10)
N1	0.0384 (13)	0.0396 (13)	0.0323 (12)	−0.0122 (10)	−0.0057 (10)	−0.0077 (10)
N2	0.0373 (13)	0.0398 (13)	0.0321 (12)	−0.0150 (10)	−0.0042 (10)	−0.0083 (10)
N3	0.0470 (16)	0.0303 (13)	0.0344 (13)	−0.0147 (11)	−0.0037 (11)	0.0015 (10)
C1	0.0291 (13)	0.0281 (13)	0.0287 (13)	−0.0080 (10)	−0.0059 (10)	−0.0067 (10)
C2	0.0380 (15)	0.0338 (14)	0.0363 (15)	−0.0084 (11)	−0.0174 (12)	−0.0037 (11)
C3	0.072 (2)	0.0444 (17)	0.0244 (14)	−0.0280 (16)	−0.0155 (13)	0.0040 (12)
C4	0.0558 (19)	0.063 (2)	0.0282 (14)	−0.0323 (16)	0.0116 (13)	−0.0178 (14)
C5	0.0375 (15)	0.0362 (15)	0.0373 (15)	−0.0087 (12)	0.0005 (12)	−0.0145 (12)
C6	0.0462 (16)	0.0307 (14)	0.0247 (13)	−0.0169 (12)	−0.0096 (11)	−0.0004 (10)
C7	0.0374 (15)	0.0374 (15)	0.0305 (14)	−0.0143 (12)	−0.0001 (11)	−0.0116 (11)
C8	0.0528 (18)	0.0266 (14)	0.0368 (15)	−0.0132 (12)	−0.0126 (13)	−0.0024 (11)
C9	0.0482 (17)	0.0484 (17)	0.0306 (14)	−0.0320 (14)	−0.0096 (12)	0.0051 (12)
C10	0.0321 (15)	0.0485 (17)	0.0370 (15)	−0.0121 (12)	−0.0116 (12)	−0.0030 (13)
C11	0.0298 (14)	0.0337 (15)	0.0360 (14)	−0.0116 (11)	−0.0057 (11)	−0.0035 (12)
C12	0.0466 (16)	0.0268 (13)	0.0241 (13)	−0.0124 (11)	−0.0073 (11)	0.0002 (10)
C13	0.0408 (15)	0.0322 (14)	0.0308 (14)	−0.0107 (12)	−0.0073 (11)	−0.0008 (11)
C14	0.0407 (15)	0.0342 (14)	0.0304 (14)	−0.0137 (12)	−0.0044 (11)	−0.0030 (11)
C15	0.0435 (15)	0.0291 (13)	0.0236 (12)	−0.0157 (11)	−0.0035 (11)	−0.0048 (10)
C16	0.0418 (15)	0.0300 (13)	0.0244 (13)	−0.0110 (11)	−0.0031 (11)	−0.0070 (10)
C17	0.0427 (16)	0.0402 (15)	0.0256 (13)	−0.0212 (12)	0.0033 (11)	−0.0096 (11)
C18	0.0426 (17)	0.0498 (18)	0.0515 (18)	−0.0149 (14)	−0.0056 (14)	0.0038 (15)
C19	0.0439 (17)	0.0366 (15)	0.0388 (16)	−0.0082 (12)	−0.0016 (12)	−0.0089 (12)
P1	0.0332 (4)	0.0384 (4)	0.0465 (4)	−0.0085 (3)	0.0011 (3)	0.0085 (3)
F1	0.076 (4)	0.119 (5)	0.139 (6)	−0.058 (4)	−0.049 (4)	0.015 (5)
F2	0.058 (3)	0.085 (4)	0.114 (4)	−0.017 (3)	−0.029 (3)	−0.036 (4)
F3	0.039 (2)	0.050 (3)	0.122 (6)	0.002 (2)	0.019 (3)	0.043 (3)
F4	0.086 (5)	0.048 (3)	0.075 (4)	0.005 (3)	0.024 (3)	0.028 (3)
F5	0.088 (6)	0.090 (5)	0.067 (3)	−0.024 (4)	0.030 (3)	−0.036 (3)
F6	0.128 (9)	0.098 (6)	0.056 (3)	−0.013 (5)	0.049 (4)	−0.026 (4)
F1A	0.155 (7)	0.152 (8)	0.096 (5)	0.018 (6)	−0.083 (5)	0.002 (5)
F2A	0.240 (9)	0.110 (6)	0.063 (4)	−0.009 (6)	−0.071 (5)	−0.002 (4)
F3A	0.185 (8)	0.060 (4)	0.099 (5)	−0.057 (4)	0.013 (5)	−0.019 (4)
F4A	0.116 (6)	0.049 (4)	0.194 (9)	−0.035 (4)	−0.003 (6)	−0.031 (6)
F5A	0.064 (5)	0.073 (6)	0.249 (14)	0.029 (4)	0.091 (7)	0.081 (7)
F6A	0.034 (3)	0.095 (7)	0.178 (11)	0.016 (3)	0.024 (5)	0.083 (7)
O3	0.0341 (12)	0.0587 (16)	0.0721 (17)	−0.0104 (11)	0.0085 (11)	0.0039 (12)

### 1'-[2-(1-Amino-2,6-dimethylphenyl)diazen-1-yl]cobaltocenium-1-carboxylic acid hexafluoridophosphate monohydrate (5) Geometric parameters (Å, º)

**Table:**

Co1—C8	2.029 (3)	C8—H8	0.9500
Co1—C1	2.029 (2)	C9—C10	1.414 (4)
Co1—C9	2.030 (3)	C9—H9	0.9500
Co1—C7	2.033 (3)	C10—H10	0.9500
Co1—C2	2.036 (3)	C12—C13	1.391 (4)
Co1—C10	2.036 (3)	C12—C17	1.402 (4)
Co1—C5	2.039 (3)	C13—C14	1.379 (3)
Co1—C4	2.041 (3)	C13—H13	0.9500
Co1—C3	2.047 (3)	C14—C15	1.415 (4)
Co1—C6	2.064 (2)	C14—C18	1.501 (4)
O1—C11	1.199 (3)	C15—C16	1.422 (4)
O2—C11	1.309 (3)	C16—C17	1.381 (3)
O2—H2O	0.837 (19)	C16—C19	1.498 (4)
N1—N2	1.263 (3)	C17—H17	0.9500
N1—C6	1.424 (3)	C18—H18A	0.9800
N2—C12	1.407 (3)	C18—H18B	0.9800
N3—C15	1.356 (3)	C18—H18C	0.9800
N3—H1N	0.878 (18)	C19—H19A	0.9800
N3—H2N	0.881 (18)	C19—H19B	0.9800
C1—C5	1.415 (4)	C19—H19C	0.9800
C1—C2	1.428 (3)	P1—F1A	1.517 (6)
C1—C11	1.486 (3)	P1—F5A	1.531 (8)
C2—C3	1.411 (4)	P1—F4A	1.534 (7)
C2—H2	0.9500	P1—F6A	1.540 (9)
C3—C4	1.411 (4)	P1—F6	1.544 (7)
C3—H3	0.9500	P1—F3	1.556 (4)
C4—C5	1.417 (4)	P1—F1	1.561 (5)
C4—H4	0.9500	P1—F2	1.562 (4)
C5—H5	0.9500	P1—F4	1.577 (5)
C6—C10	1.424 (4)	P1—F2A	1.579 (5)
C6—C7	1.425 (4)	P1—F5	1.585 (8)
C7—C8	1.419 (4)	P1—F3A	1.592 (6)
C7—H7	0.9500	O3—H3A	0.856 (19)
C8—C9	1.404 (4)	O3—H3B	0.835 (19)
			
C8—Co1—C1	144.40 (11)	C8—C7—Co1	69.40 (15)
C8—Co1—C9	40.48 (12)	C6—C7—Co1	70.80 (15)
C1—Co1—C9	174.67 (11)	C8—C7—H7	126.2
C8—Co1—C7	40.90 (10)	C6—C7—H7	126.2
C1—Co1—C7	113.95 (10)	Co1—C7—H7	125.2
C9—Co1—C7	68.65 (11)	C9—C8—C7	108.5 (2)
C8—Co1—C2	113.50 (11)	C9—C8—Co1	69.78 (16)
C1—Co1—C2	41.12 (10)	C7—C8—Co1	69.70 (15)
C9—Co1—C2	143.37 (11)	C9—C8—H8	125.8
C7—Co1—C2	109.64 (11)	C7—C8—H8	125.8
C8—Co1—C10	68.40 (12)	Co1—C8—H8	126.3
C1—Co1—C10	135.01 (11)	C8—C9—C10	108.4 (2)
C9—Co1—C10	40.70 (11)	C8—C9—Co1	69.74 (15)
C7—Co1—C10	68.79 (11)	C10—C9—Co1	69.90 (15)
C2—Co1—C10	175.35 (11)	C8—C9—H9	125.8
C8—Co1—C5	174.07 (11)	C10—C9—H9	125.8
C1—Co1—C5	40.70 (10)	Co1—C9—H9	126.1
C9—Co1—C5	134.61 (12)	C9—C10—C6	108.0 (2)
C7—Co1—C5	144.43 (11)	C9—C10—Co1	69.39 (15)
C2—Co1—C5	68.73 (11)	C6—C10—Co1	70.71 (14)
C10—Co1—C5	109.82 (12)	C9—C10—H10	126.0
C8—Co1—C4	134.27 (11)	C6—C10—H10	126.0
C1—Co1—C4	68.36 (10)	Co1—C10—H10	125.5
C9—Co1—C4	109.53 (11)	O1—C11—O2	125.5 (2)
C7—Co1—C4	174.18 (12)	O1—C11—C1	122.3 (2)
C2—Co1—C4	68.28 (12)	O2—C11—C1	112.2 (2)
C10—Co1—C4	113.72 (12)	C13—C12—C17	118.9 (2)
C5—Co1—C4	40.63 (11)	C13—C12—N2	114.1 (2)
C8—Co1—C3	109.59 (11)	C17—C12—N2	127.1 (2)
C1—Co1—C3	68.37 (10)	C14—C13—C12	122.1 (3)
C9—Co1—C3	113.47 (11)	C14—C13—H13	119.0
C7—Co1—C3	134.63 (12)	C12—C13—H13	119.0
C2—Co1—C3	40.43 (11)	C13—C14—C15	118.3 (2)
C10—Co1—C3	143.69 (12)	C13—C14—C18	121.2 (3)
C5—Co1—C3	68.24 (12)	C15—C14—C18	120.4 (2)
C4—Co1—C3	40.39 (13)	N3—C15—C14	119.8 (2)
C8—Co1—C6	68.24 (11)	N3—C15—C16	119.4 (2)
C1—Co1—C6	110.41 (10)	C14—C15—C16	120.8 (2)
C9—Co1—C6	68.23 (10)	C17—C16—C15	118.3 (2)
C7—Co1—C6	40.71 (11)	C17—C16—C19	121.7 (2)
C2—Co1—C6	135.43 (11)	C15—C16—C19	120.0 (2)
C10—Co1—C6	40.65 (10)	C16—C17—C12	121.6 (2)
C5—Co1—C6	114.40 (11)	C16—C17—H17	119.2
C4—Co1—C6	144.43 (13)	C12—C17—H17	119.2
C3—Co1—C6	174.79 (12)	C14—C18—H18A	109.5
C11—O2—H2O	115 (3)	C14—C18—H18B	109.5
N2—N1—C6	109.2 (2)	H18A—C18—H18B	109.5
N1—N2—C12	114.9 (2)	C14—C18—H18C	109.5
C15—N3—H1N	120 (2)	H18A—C18—H18C	109.5
C15—N3—H2N	119 (2)	H18B—C18—H18C	109.5
H1N—N3—H2N	121 (3)	C16—C19—H19A	109.5
C5—C1—C2	108.0 (2)	C16—C19—H19B	109.5
C5—C1—C11	123.9 (2)	H19A—C19—H19B	109.5
C2—C1—C11	128.0 (2)	C16—C19—H19C	109.5
C5—C1—Co1	70.02 (14)	H19A—C19—H19C	109.5
C2—C1—Co1	69.69 (14)	H19B—C19—H19C	109.5
C11—C1—Co1	124.91 (18)	F1A—P1—F5A	90.6 (6)
C3—C2—C1	107.6 (2)	F1A—P1—F4A	98.4 (6)
C3—C2—Co1	70.20 (15)	F5A—P1—F4A	88.8 (6)
C1—C2—Co1	69.19 (14)	F1A—P1—F6A	86.0 (6)
C3—C2—H2	126.2	F5A—P1—F6A	174.5 (9)
C1—C2—H2	126.2	F4A—P1—F6A	87.4 (6)
Co1—C2—H2	126.0	F6—P1—F3	89.5 (5)
C2—C3—C4	108.4 (2)	F6—P1—F1	95.1 (6)
C2—C3—Co1	69.37 (15)	F3—P1—F1	88.6 (4)
C4—C3—Co1	69.60 (16)	F6—P1—F2	84.5 (5)
C2—C3—H3	125.8	F3—P1—F2	90.2 (4)
C4—C3—H3	125.8	F1—P1—F2	178.8 (3)
Co1—C3—H3	126.8	F6—P1—F4	88.3 (5)
C3—C4—C5	108.3 (2)	F3—P1—F4	177.6 (5)
C3—C4—Co1	70.01 (16)	F1—P1—F4	90.6 (4)
C5—C4—Co1	69.60 (15)	F2—P1—F4	90.5 (4)
C3—C4—H4	125.9	F1A—P1—F2A	174.7 (5)
C5—C4—H4	125.9	F5A—P1—F2A	88.1 (7)
Co1—C4—H4	126.1	F4A—P1—F2A	86.7 (4)
C1—C5—C4	107.7 (2)	F6A—P1—F2A	95.6 (6)
C1—C5—Co1	69.27 (14)	F6—P1—F5	177.1 (6)
C4—C5—Co1	69.78 (16)	F3—P1—F5	91.9 (5)
C1—C5—H5	126.1	F1—P1—F5	87.5 (5)
C4—C5—H5	126.1	F2—P1—F5	92.9 (4)
Co1—C5—H5	126.4	F4—P1—F5	90.4 (4)
C10—C6—N1	121.0 (2)	F1A—P1—F3A	84.8 (4)
C10—C6—C7	107.5 (2)	F5A—P1—F3A	87.5 (6)
N1—C6—C7	131.5 (2)	F4A—P1—F3A	175.2 (4)
C10—C6—Co1	68.64 (14)	F6A—P1—F3A	96.4 (5)
N1—C6—Co1	127.67 (18)	F2A—P1—F3A	90.0 (5)
C7—C6—Co1	68.48 (14)	H3A—O3—H3B	103 (4)
C8—C7—C6	107.6 (2)		
			
C6—N1—N2—C12	177.3 (2)	C7—C8—C9—Co1	−59.15 (18)
C5—C1—C2—C3	−0.2 (3)	C8—C9—C10—C6	1.1 (3)
C11—C1—C2—C3	−179.0 (2)	Co1—C9—C10—C6	60.46 (18)
Co1—C1—C2—C3	−59.96 (18)	C8—C9—C10—Co1	−59.34 (19)
C5—C1—C2—Co1	59.76 (18)	N1—C6—C10—C9	178.3 (2)
C11—C1—C2—Co1	−119.0 (3)	C7—C6—C10—C9	−2.1 (3)
C1—C2—C3—C4	0.5 (3)	Co1—C6—C10—C9	−59.63 (19)
Co1—C2—C3—C4	−58.8 (2)	N1—C6—C10—Co1	−122.0 (2)
C1—C2—C3—Co1	59.32 (18)	C7—C6—C10—Co1	57.55 (18)
C2—C3—C4—C5	−0.6 (3)	C5—C1—C11—O1	4.2 (4)
Co1—C3—C4—C5	−59.28 (19)	C2—C1—C11—O1	−177.2 (3)
C2—C3—C4—Co1	58.70 (19)	Co1—C1—C11—O1	92.3 (3)
C2—C1—C5—C4	−0.2 (3)	C5—C1—C11—O2	−175.4 (2)
C11—C1—C5—C4	178.7 (2)	C2—C1—C11—O2	3.3 (4)
Co1—C1—C5—C4	59.40 (19)	Co1—C1—C11—O2	−87.2 (3)
C2—C1—C5—Co1	−59.55 (17)	N1—N2—C12—C13	177.0 (2)
C11—C1—C5—Co1	119.3 (2)	N1—N2—C12—C17	−3.4 (4)
C3—C4—C5—C1	0.5 (3)	C17—C12—C13—C14	1.6 (4)
Co1—C4—C5—C1	−59.08 (18)	N2—C12—C13—C14	−178.7 (2)
C3—C4—C5—Co1	59.5 (2)	C12—C13—C14—C15	−0.7 (4)
N2—N1—C6—C10	176.2 (2)	C12—C13—C14—C18	178.0 (3)
N2—N1—C6—C7	−3.3 (4)	C13—C14—C15—N3	179.5 (2)
N2—N1—C6—Co1	90.4 (3)	C18—C14—C15—N3	0.8 (4)
C10—C6—C7—C8	2.3 (3)	C13—C14—C15—C16	−0.8 (4)
N1—C6—C7—C8	−178.2 (3)	C18—C14—C15—C16	−179.5 (3)
Co1—C6—C7—C8	59.90 (18)	N3—C15—C16—C17	−179.0 (2)
C10—C6—C7—Co1	−57.65 (18)	C14—C15—C16—C17	1.3 (4)
N1—C6—C7—Co1	121.9 (3)	N3—C15—C16—C19	0.9 (4)
C6—C7—C8—C9	−1.6 (3)	C14—C15—C16—C19	−178.8 (2)
Co1—C7—C8—C9	59.20 (19)	C15—C16—C17—C12	−0.3 (4)
C6—C7—C8—Co1	−60.79 (18)	C19—C16—C17—C12	179.8 (2)
C7—C8—C9—C10	0.3 (3)	C13—C12—C17—C16	−1.1 (4)
Co1—C8—C9—C10	59.45 (19)	N2—C12—C17—C16	179.3 (2)

### 1'-[2-(1-Amino-2,6-dimethylphenyl)diazen-1-yl]cobaltocenium-1-carboxylic acid hexafluoridophosphate monohydrate (5) Hydrogen-bond geometry (Å, º)

**Table:**

*D*—H···*A*	*D*—H	H···*A*	*D*···*A*	*D*—H···*A*
N3—H2*N*···O1^i^	0.88 (2)	2.18 (2)	3.015 (3)	159 (3)
N3—H1*N*···F5	0.88 (2)	2.29 (3)	2.994 (10)	137 (3)
N3—H1*N*···F5*A*	0.88 (2)	2.24 (3)	2.896 (8)	131 (3)
O2—H2*O*···O3	0.84 (2)	1.80 (2)	2.625 (3)	170 (4)
O3—H3*A*···N1^ii^	0.86 (2)	2.06 (2)	2.907 (3)	171 (4)
O3—H3*B*···F5^iii^	0.84 (2)	2.22 (3)	2.988 (8)	153 (4)
O3—H3*B*···F2*A*^iii^	0.84 (2)	2.34 (3)	3.112 (8)	154 (4)

Symmetry codes: (i) −*x*+1, −*y*+2, −*z*+1; (ii) *x*+1, *y*, *z*; (iii) −*x*+2, −*y*+2, −*z*+1.
